# Endoscopic dacryocystorhinostomy with an otologic T-type ventilation tube in repeated revision cases

**DOI:** 10.1186/s12886-017-0539-7

**Published:** 2017-08-07

**Authors:** Sihai Wu, Ting Xu, Bin Fan, Dajiang Xiao

**Affiliations:** Department of Otorhinolarynogology, The Second People’s Hospital of Wuxi, Nanjing Medical University, NO. 68, Zhongshan Road Wuxi, Nanjing, Jiangsu 214002 China

**Keywords:** Endoscopic dacryocystorhinostomy, T-type ventilation tube, Chronic dacryocystitis, Clinical efficacy

## Abstract

**Background:**

To compare the frequency of appearance of complications, anatomical success and functional success after conventional endoscopic dacryocystorhinostomies (EN-DCRs) or EN-DCR with otologic T-Type ventilation tube combined with silicone tube intubation in repeated revision cases.

**Methods:**

Twenty-two patients who had epiphora and recurrent dacryocystitis after at least a previous failed revision DCR as well as 22 patients receiving conventional EN-DCR only were enrolled in the study between January 2008 and December 2011. Operations were performed by using an otologic T-tube combined with silicone tube intubation. Oral antibiotics, nasal steroids, oral antihistamines, and antibiotic eyedrops were given to all cases. The ventilation tubes were removed 6 to 20 weeks after surgery.

**Results:**

Of 22 cases, all cases achieved anatomical success, 19 cases were symptom free, and 3 cases had decreased continuation in complications with a functional success rate of 81.8%. The overall success rates were significantly higher than those in patients undertaking conventional EN-DCR only (*P* < 0.01).

**Conclusion:**

The revision endoscopic DCR has a high rate of failure. The usage of a T-type ventilation tube can significantly improve the success rate of surgery.

**Trial registration number:**

ChiCTR-INR-17012160, retrospectively registered on July 27th, 2017.

## Background

It has been reported that endonasal dacryocystorhinostomies (EN-DCRs) yields good esthetic, functional results and similar success rate to that of the external dacryocystorhinostomies (EX-DCRs) [1–5]. By simulating EX-DCRs in EN-DCRs (mainly in two aspects: 1 maximizing bone window and complete exposure of the inside wall of larimal sac; 2 Ensuring anatomical overlap of the lacrimal sac and nasal mucosal flap), the EN-DCRs can achieve similar successful rates to EX-DCRs [6, 7]. But for the cases of small sac who have lacrimal sac mucosa fibrosis and mucosa scarring resulting from limited residual sac mucosa caused by previous DCRs, the success rate of revision DCR is significantly reduced regardless of the surgical approaches [8, 9]. One of the most important reasons is that it is difficult to form lacrimal sac mucosal flap and nasal mucosa flap anatsatomosis, which causes stoma scarring and resultant surgical failure. Those patients often require repeated revision DCRs, and the revision DCRs often require special surgical approach such as agger nasi cell mucosal autograft for lacrimal sac reconstruction or mucosal grafting [10, 11]. A recent retrospective review has reported that powered EN-DCR is a suitable option for revising failed DCRs in which yields good long-term results [3]. Inserting silicone tubes through the inferior and the superior puncta significantly improved the successful rate of revision DCR [2, 4]. However, silicone tubes were too thin to induce the closure of newly performed ostia for patients with the risk factors of failure. To improve the current EN-DCR procedure, we have inserted an otologic T-type tube in addition to silicone tube into the lacrimal sac cavity., and we have achieved a higher success rate than conventional EN-DCR with careful treatment after surgery.

## Methods

### Patients

This is a prospective study where 22 patients who had previously undergone at least once failed EN-DCR were enrolled in our study during January 2008 to December 2011. The main symptoms of all patients were epiphora. All patients were given a detailed ophthalmic and otolaryngologic examination preoperatively. The former included lacrimal probe and rinse to eliminate canalicular and tear duct obstruction. The latter included a detailed nasal endoscopy to determine whether there were nasal abnormalities such as high deviated septum, which might influence the surgery effect [12]. All the operations, including the last revision DCR, were performed by the same surgeon. In addition, 22 patients who received conventional EN-DCR only were included as control.

The study was approved by the Ethical Committee of The Second People’s Hospital of Wuxi. Informed consents were obtained from all individuals included in the study.

### Surgical technique

**Fig. 1:**
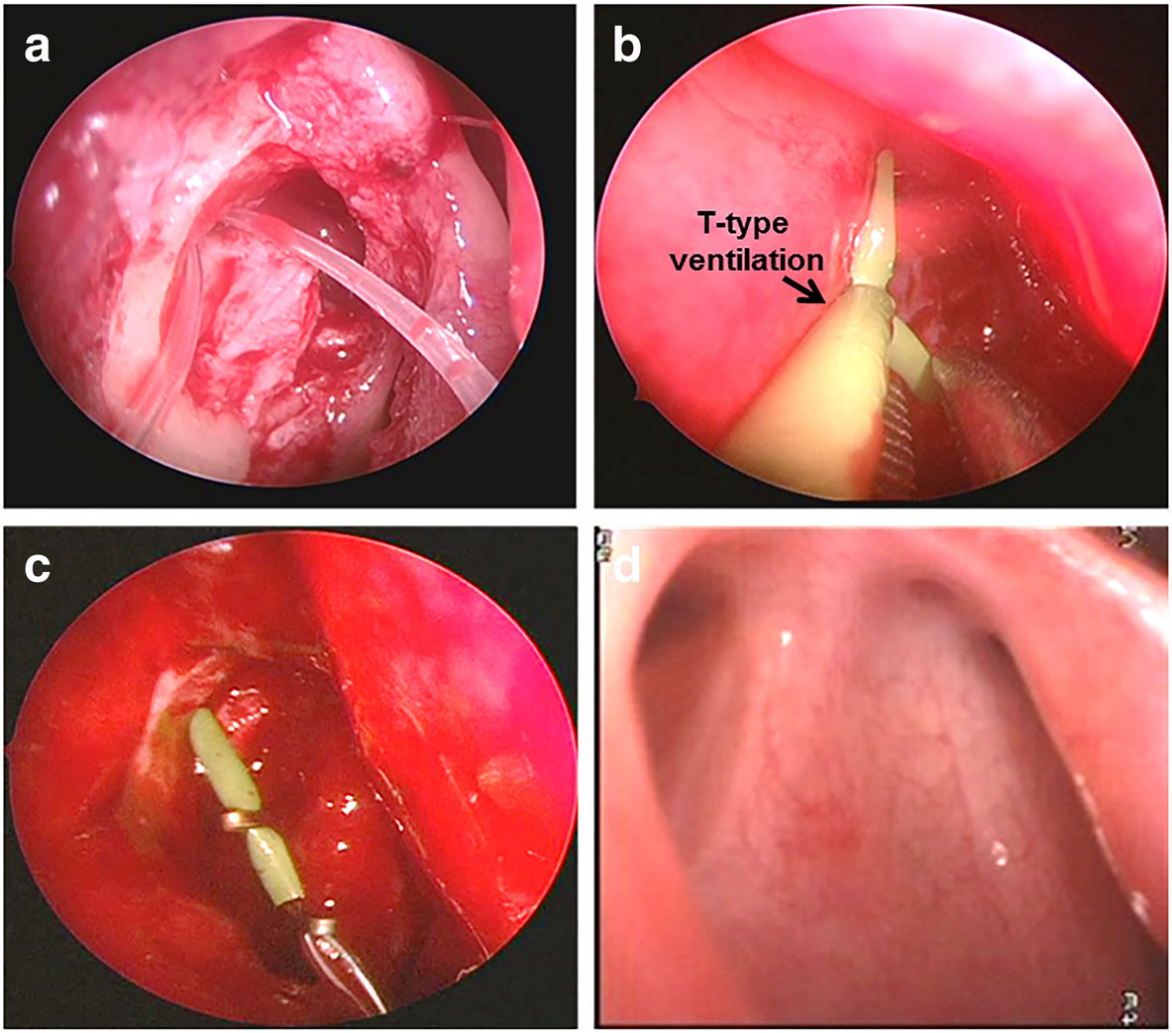
Surgical techniques. **a** Silicone tube needles inserted through the inferior and the superior puncta grasped in the nasal cavity (*right*). **b** Insertion of the T-type ventilation tube (*black arrow*) using micro-alligator forceps. T-type ventilation tube was set out of the silicone tube. **c** A gun-shaped clamp was used to make the T-type tube gradually sliding towards the sac. **d** The ostium status in referral visit after removing T-tube

EN-DCR was performed under general anesthesia. After decongestion and vasoconstriction of the nasal mucosa with packing of 1/1000 adrenaline solution, an elbow probe was used to determine the exact location of the last bone window. Under endoscopic visualization with a 30° endoscope, a curved mucosal flap incision was carried 0.5 cm more anteriorly over the front edge of bone window. When necessary, the bone window was enlarged with a 15° medtronic sac drill until complete sac exposure was obtained including inside wall. A lacrimal probe was inserted through the inferior punctum into sac, the median wall of the sac was pushed into the nasal cavity. The medial 1*1 cm section of the median wall of the lacrimal sac was excised upon the mucosal incision with the sickled blade. Silicone tube needles which were inserted through the inferior and the superior puncta were grasped in the nasal cavity, followed by the insertion of the T-type ventilation tube using micro-alligator forceps, which were commonly used in ear surgery (Fig. 1). T-type ventilation tube was set out of the silicone tube when assistant pulled the two ends of the silicone tube. A gun-shaped clamp was used to make the T-type tube gradually sliding towards the sac. Two feet of T-tube was unfolded into sac with probes and was clipped with titanium clips to prevent it from falling off. Normal mucosa was reset and make sure there was no active bleeding. One patient who underwent deviated septum surgery was given nasal packing, and the rest were given no nasal packing.

### Postoperative disposition

Postoperatively, the nasal packing material was removed 2 days later, and the patients were given oral antibiotics for 7 days, antibiotic eyedrops for 15 days, nasal steroids for 3 weeks, and oral antihistamines for a week. Patients were followed up every 2 weeks to report any symptoms, to remove scars, granulation tissue, and possible synechiae under endoscopy until the occurrence of complete local epithelialization. Check whether the T-tube prolapse out of sac and reset the tube if it prolapses. If no partial granulation or adhesion, and smooth stoma was observed in 2 consecutive endoscopies, T-tube was removed. To remove T-tube, silicone tube was cut at innur canthus, and then the T-tube was removed together with the silicone tube under endoscopy. Lacrimal irrigation was carried out weekly for the first month, and twice a month since then. The evaluation of the success was conducted at two time points: 1, disappearance of patients’ symptoms; 2, no reflux in lacrimal irrigation.

### Statistical analyses

Statistical analyses were performed using GraphPad Prism v5.0 (Graphpad Software Inc.). Two sample test or Chi-square test was used for comparison of qualitative data. A *p* < 0.05 was considered to be statistically significant.

## Results

Modified revision EN-DCR was performed on 22 patients (7 male and 15 female), and the average age for those patients at the time of revision was 44.5 years (range from 26 to 68 years; SD, 15.1). Of those patients, 15 cases have previously undergone revision EN-DCRs twice, and 2 cases have undergone revision EN-DCRs three times. The patients have previously undergone either EX-DCR or EN-DCR, but the latest surgery of all patients was EN-DCR. The time from last failed DCR to recurrence of epiphora varied from 5 to 37 months with a mean time of 12 months. The main causes of previous failure were membranous scarring over ostium (8/22), ostium closure (5/22), or wall of lacrimal sac thickness and fibrosis (9/22) (Table 1). In parallel, 22 patients who received conventional EN-DCR were included as a control group. There were no significant differences in age or gender distribution between the experimental and control groups (Table 2). Of these patients, 18 cases have previously undergone revision EN-DCRs twice and 1 case has undergone revision EN-DCRs three times. The time from last failed DCR to recurrence of epiphora varied from 6 to 35 months with a mean time of 12.5 months (Table 3).

**Table 1:** General characteristics of patients receiving modified revision EN-DCR

Case	Side	Type of failed DCR and year	Time from last failed DCR to recurrence of epiphora (months)	Cause(s) of failure	The times of tube prolapse	the duration of tube intubation(weeks)
1	L	EX 2006 EN 2007	15	Membranous scarring over ostium	0	6
2	R	EX 2004 EN 2006	18	Wall of lacrimal sac thickness and fibrosis	1	8
3	R	EX 2002 EN 2005	37	Ostium closure	0	15
4	L	EX 2003 EX 2005 EN 2008	25	Membranous scarring over ostium	0	20
5	L	EN 2007 EN 2008	8	Ostium closure	1	14
6	R	EX 2007 EN 2009	7	Wall of lacrimal sac	0	18
7	R	EX 2006 EX 2008 EN 2010	10	Membranous scarring over ostium	2	8
8	L	EN 2009 EN 2010	6	Membranous scarring over ostium	0	10
9	L	EX 2009	9	Ostium closure	3	13
10	R	EN 2009 EN 2010	5	Wall of lacrimal sac thickness and fibrosis	0	14
11	L	EN 2008 EN 2009	12	Wall of lacrimal sac thickness and fibrosis	0	18
12	R	EN 2009	8	Wall of lacrimal sac thickness and fibrosis	0	15
13	R	EN 2003 EN 2005	10	Ostium closure	2	20
14	L	EX 2007 EN 2009	12	Membranous scarring over ostium	3	10
15	L	EX 2008	6	Membranous scarring over ostium	2	15
16	L	EN 2005 EN 2009	5	Wall of lacrimal sac	3	13
17	L	EX 2008 EN 2010	8	Ostium closure	1	12
18	R	EN 2004 EN 2009	10	Wall of lacrimal sac	1	11
19	R	EN 2005 EN 2008	13	Membranous scarring over ostium	2	8
20	R	EX 2005 EN 2008	16	Membranous scarring over ostium	3	6
21	L	EX 2006 EN 2008	9	Wall of lacrimal sac	2	6
22	L	EN 2009	7	Wall of lacrimal sac	0	12

Age range: 26–68; Number of male: 7; Number of female: 15

**Table 2:** Comparison of demographics of modified revision EN-DCR and conventional ENDCR Groups

Variables	Modified EN-DCR	EN-DCR	*P* Value
Age (yrs)			
Mean	44.5	46.3	> 0.8^a^
Standard deviation	15.1	16.3	
Gender, n (%)			
Male	7 (31.8)	10 (45.4)	> 0.8^b^
Female	15 (68.2)	12 (54.6)	

^a^t test

^b^Chi-square test

**Table 3:** General characteristics of patients receiving conventional revision EN-DCR

Case	Side	Type of failed DCR and year	Time from last failed DCR to recurrence of epiphora (months)	Cause(s) of failure
1	R	EX 2006 EX 2007	16	Ostium closure
2	R	EX 2004 EN 2006	22	Wall of lacrimal sac thickness and fibrosis
3	L	EN 2002 EX 2005	19	Ostium closure
4	R	EX 2003 EN 2008	25	Membranous scarring over ostium
5	L	EX 2007 EN 2008	35	Ostium closure
6	L	EX 2007 EN 2009	14	Wall of lacrimal sac
7	R	EN 2006 EX 2008 EN 2006	12	Membranous scarring over ostium
8	L	EX 2009 EN 2010	8	Membranous scarring over ostium
9	R	EX 2009 EN 2009	7	Wall of lacrimal sac thickness and fibrosis
10	R	EN 2009 EN 2010	9	Wall of lacrimal sac thickness and fibrosis
11	L	EN 2008 EN 2009	12	Ostium closure
12	L	EN 2009	12	Wall of lacrimal sac thickness and fibrosis
13	R	EN 2003 EN 2005	11	Wall of lacrimal sac
14	R	EX 2007 EN 2009	6	Ostium closure
15	R	EX 2008	8	Membranous scarring over ostium
16	L	EN 2005 EN 2009 EX 2008	7	Wall of lacrimal sac
17	L	EX 2008 EN 2010	9	Ostium closure
18	L	EN 2004 EN 2007	11	Wall of lacrimal sac
19	R	EN 2008 EN 2006	12	Membranous scarring over ostium
20	L	EX 2007 EN 2009	14	Wall of lacrimal sac
21	R	EX 2008 EN 2009	8	Membranous scarring over ostium
22	L	EN 2007	7	Wall of lacrimal sac

Age range: 31–66; Number of male: 8; Number of female: 14

All patients felt the symptom of epiphora, nasal secretions and foreign body sensation. However, these symptoms disappeared after the T-tubes were removed. In the postoperative follow-up visits, 8 patients reported alleviated epiphora before the tube was removed, and 6 patients reported foreign body sensation in nasal cavity or inner canthus with increased nasal secretion. In referral, T-tube was found prolapsed in 9 patients among whom 5 cases prolapsed once, 3 cases prolapsed twice, 1 case prolapsed three times. The tubes were successfully reset whenever prolapses were observed. The duration of tube intubation varied from 6 to 20 weeks, with a mean time of 12.5 weeks. The follow-up time varied from 2 to 3 years with a mean time of 2.3 years. After removing T-tube, 3 patients reported mild epiphora when they felt fatigue in cold weather, but the symptoms disappeared after eyedrops and lacrimal. In the group who underwent modified EN-DCR, 3 patients had complications with 2 cases with eyelid edema which disappeared 1 week after operation and 1 case with appearance of granulation tissue formation surrounding the T-tubes which were cleared under endoscope. In most patients, crusts were found to be formed surround the T-tubes, which were cleared and cleaned with nasal saline in follow-up visits. Eyelid edema and bleeding spots typically recovered 1 week after surgery without interference. In the patients undergoing conventional DCR, 6 patients suffered from eyelid edema and 8 patients reported bleeding spots, and 4 patients experienced epiphora, significantly higher than modified EN-DCR (90.0% vs 13.6%, *P* < 0.0001) (Table 4).

**Table 4:** Appearance of complications in modified revision EN-DCR and conventional ENDCR Groups

Complications	Modified EN-DCR N (%)	EN-DCR N (%)	*P* Value
Present	3 (13.6)	20 (90.9)	<0.0001^a^
Absent	19 (86.4)	2 (9.1)	

^a^Chi-square test

Table 5 summarized surgical outcomes 2 years after surgery. Anatomical success was achieved in 100% (22/22) of modified EN-DCR and 40.9% (9/22) of conventional EN-DCRs. Functional success was observed in 90.9.0% (20/22) of modified EN-DCR and 22.7% (5/22) of EN-DCR. The difference in surgical outcomes between these two surgeries was statistically significant with a two-sample test for equality of proportions with continuity correction (*P* < 0.01). With 44 study patients, the power of this test is 80% with α = 0.05 to detect a clinically significant decrease in the success rate of 8%.

**Table 5:** Outcome for modified revision EN-DCR and conventional EN-DCR Groups

Variables	Modified EN-DCR N (%)	EN-DCR N (%)	*P* Value
Anatomical success	22 (100)	9 (40.9)	<0.01^a^

^a^Two-sample test for equality of proportions with continuity correction; Power = 80% for α = 0.05 to detect a decreased success rate of 8%

## Discussion

In the present study, we slightly modified the approach where an otologic T-tube combined with silicone tube intubation was applied in EN-DCRs. Compared with silicone tube intubation only, this combined approach significantly improved the successful rate. This improvement may be due to the fact that T-tube acts as a support for residual sac mucosa, and it promotes the anastomosis of nasal and lacrimal sac mucosa, which results in spacious and long-lasting channel formation between the sac and nasal cavity, thus easing the ostium stenosis and closure caused by gradual contraction. No severe complications, such as orbital injury, occurred during operation for any of the patients, the incidence of minor postoperative complications such as eyelid edema and bleeding spots were lower than the control group, who have only undergone conventional EN-DCR by the same surgeon.

The closure of newly created ostium is the common cause of failure. It is generally accepted that the size of the excised lacrimal bone and created ostium is important for the long-term outcomes [5]. If the bone window is large enough, the sac is fully exposed, and the anastomosis of nasal and lacrimal sac mucosa flaps is fully achieved, most revision DCRs do not require additional T-tube implants since it prolongs the operation time and adds on additional expense for patients. Moreover, some patients may feel discomfort in the nose and inner canthus caused by T-tube implants. But for patients with high risk of failure such as those with small sac, lacrimal sac mucosa fibrosis and scarring or limited residual sac mucosa, T-tube combined with silicone tube intubation is a better alternative than conventional EN-DCRs.

## Conclusions

Our modified procedure achieved both anatomical success rate and functional success rate with lower occurrence of minor complications. The functional success rate was lower than the anatomical success rate probably due to systolic dysfunction of the remnants of lacrimal sac and the poor siphon function of lacrimal point. While we appreciate the moderate success rate of current EN-DCR, we believe that for the revision EN-DCR, T-tube combined with silicone tube intubation needs to be improved due to the two reasons: firstly, it is needed to establish some objective indicators for the combined intubation; secondly, the otologic T-tube we applied was used for the treatment of secretory otitis media but not specifically for DCR. We believe if we can further optimize the material and structure of T-tube to make it more suitable for the application in the lacrimal sac, there will be more widely usage of T-tube in DCR.

## Abbreviation

EN-DCRs: Endoscopic dacryocystorhinostomies
